# Graph-based signal integration for high-throughput phenotyping

**DOI:** 10.1186/1471-2105-13-S13-S2

**Published:** 2012-08-24

**Authors:** Jorge R Herskovic, Devika Subramanian, Trevor Cohen, Pamela A Bozzo-Silva, Charles F Bearden, Elmer V Bernstam

**Affiliations:** 1 School of Biomedical Informatics, The University of Texas Health Science Center at Houston, Houston, TX, USA; 2 Department of Computer Science, Rice University, Houston, TX, USA; 3 Escuela de Medicina, Universidad de Los Andes, Santiago, Chile; 4 Medical School, The University of Texas Health Science Center at Houston, Houston, TX, USA

## Abstract

**Background:**

Electronic Health Records aggregated in Clinical Data Warehouses (CDWs) promise to revolutionize Comparative Effectiveness Research and suggest new avenues of research. However, the effectiveness of CDWs is diminished by the lack of properly labeled data. We present a novel approach that integrates knowledge from the CDW, the biomedical literature, and the Unified Medical Language System (UMLS) to perform high-throughput phenotyping. In this paper, we automatically construct a graphical knowledge model and then use it to phenotype breast cancer patients. We compare the performance of this approach to using MetaMap when labeling records.

**Results:**

MetaMap's overall accuracy at identifying breast cancer patients was 51.1% (n=428); recall=85.4%, precision=26.2%, and F_1_=40.1%. Our unsupervised graph-based high-throughput phenotyping had accuracy of 84.1%; recall=46.3%, precision=61.2%, and F_1_=52.8%.

**Conclusions:**

We conclude that our approach is a promising alternative for unsupervised high-throughput phenotyping.

## Background

Electronic Health Records (EHR) collect patient data obtained in the course of clinical care. These records, when aggregated in Clinical Data Warehouses (CDWs), are a rich source of data for research. For example, we may want to estimate disease prevalence, track infectious diseases, identify unexpected side-effects of drugs, identify cohorts of patients for studies and compare the effectiveness of alternative treatments for a given condition.

Unfortunately, CDWs built from EHRs have not lived up to these hopes. The fundamental problem is that we are attempting to use EHR data for purposes other than supporting clinical care. Over 20 years ago, van der Lei warned against this practice and proposed that “Data shall be used only for the purpose for which they were collected.” [1] For example, ICD-9-CM codes are routinely assigned to a patient for billing purposes, but billing rules are not meant to preserve and encode clinical reality. Instead, billing rules are meant to comply with the byzantine, sometimes mutually incompatible requirements of insurers, administrators, and regulators. For example, patients and their insurers are billed only for conditions for which they are treated by providers. In multiple-provider situations this means that each provider only sees a part of the patients’ conditions.

For example, consider a patient P with breast cancer that gets her oncological treatment at Cancer Center A. Cancer Center A bills the patient, and the patient’s insurance, for cancer care. The same patient gets tonsillitis and decides to go to outpatient clinic B. Clinic B sees her, treats her, and bills her insurance for tonsillitis. At this point in time, Cancer Center A has a record for a patient with breast cancer, and Clinic B has a record for a patient with tonsillitis.

This state of affairs is appropriate for routine clincal care. Somewhere in the patient’s record at Clinic B a physician or nurse will have written that the patient also had breast cancer. If another physician at Clinic B needs to know, she can find out by reading the patient’s file.

Now consider a researcher at Clinic B who wants to know if breast cancer predisposes people towards tonsillitis. Any attempt to find a correlation using billing data will miss patient P. The same is true for researchers trying to perform genomics research on these diseases; they will simply miss these patients.

This is not idle speculation; at our outpatient clinic, approximately 52% of patients who have or had breast cancer according to their own charts have been billed for the condition. Similarly, 23% of patients with endometrial cancer have a billing code compatible with endometrial cancer [2]. Data from other institutions and other conditions are similar. For example, 52% of patients with an ICD-9-CM code for Wegener’s Granulomatosis at St. Alexius Medical Center actually met the diagnostic criteria for the condition [3]. A strategy combining different ICD-9 codes yielded an 88% positive predictive value (PPV) for Lupus Nephritis cases at Brigham & Women’s Hospital in Boston. However, the sensitivity was impossible to compute (i.e., it was not known how many cases were missed) [4]. Other studies had similar outcomes [5-10].

Many research efforts such as those focused on Comparative Effectiveness Research (CER), genomics, proteomics, genealogy require accurate knowledge of the patient’s entire medical history and list of conditions; sometime referred to as the patient’s phenotype. These research endeavors aren’t interested in the patient’s billing history. They are interested in what conditions the patients actually had. This is also known as high-throughput phenotyping.

This information is often available in physicians’ and nurses’ notes. Further, clinical notes will contain information about past events, unlike other sources of information. For example, a patient with a remote past history of breast cancer, now without evidence of disease, will not receive medications, and will not have procedures or lab exams done that could point to the diagnosis. Clinical notes may also be more abundant than other sources of information. Our CDW contains 295,000 patients with at least one clinical note; 161,000 patients with at least one recorded vital sign; 143,000 patients with at least one medication in a structured field; and 138,000 patients with at least one lab exam. Thus, clinical notes are an important resource for research projects that require clinical information.

Manual review of hundreds of thousands of charts is impractical. Even smaller-scale manual review is expensive, and prone to error and inconsistent coding [11]. The biomedial informatics reserch community therefore continuously seeks ways to extract computable information from free text [12]. Automated coding systems such as MetaMap [13], cTAKES [14], and MedLEE [15] can map text to Unified Medical Language System (UMLS) concepts; however, without the addition of customized rules they draw no inferences from it. Many interesting problems require determining the state of the patient – i.e. “did the patient ever have breast cancer?” instead of the easier “does this document mention breast cancer?” Automated classification systems built using Weka[16] or MAVERIC’s ARC [17] address the second need, and perform very well on cross-validation [2,17]. However, these systems have two weaknesses. The first weakness is that they require training data, which are expensive and slow to create, as it requires a clinician to read each patient’s chart and decide whether the patient had the condition in question. The second weakness is that a system that works well to identify one concept may not work as well to identify a different concept, or even the same concept in another data set; in other words, these systems are not generalizable [12].

We therefore set out to build a high-throughput phenotyping system that required neither training data nor customized disease-specific rules, used available external knowledge, and performed well compared to existing automated coding systems. We based our design on the intuition that clinicians first look for explicit statements that assert that a patient has the condition of interest. If they fail to find these statements, they look for evidence of the condition. For example, the question “does the patient have diabetes?” can be answered by finding a statement in the notes that asserts that the patient has diabetes. However, if the explicit assertion is missing, it is still possible to determine whether the patient has diabetes by looking for concepts that are commonly associated with diabetes. Thus, a physician might read the chart and discover that the patient had high glycosylated hemoglobin (a lab marker of long-term glucose concentration in blood), takes metformin (a drug used to treat diabetes), and had a foot exam (commonly performed on patients with diabetes during office visits). The presence or absence these additional elements may add evidence for or against a diagnosis of diabetes respectively, in the event that the concept is explicitly mentioned. In other words, human experts use background knowledge to understand text; specifically, they look for consistency between multiple concepts found in the text.In many respects, this process mirrors the construction and integration phases of Kintsch’s influential construction-integration model of text comprehension: concepts derived from both the reader’s background knowledge and elements of the text itself are integrated during the process of constructing a mental representation of the text, and the extent to which these concepts are collectively consistent with a particular interpretation (e.g., patient has diabetes) determines whether or not this interpretation prevails.

We imitate this comprehension process [18] by constructing a nearest neighbor graph using a limited breadth-first search from a seed term on UMLS concepts extracted from our CDW, to simulate associative retrieval of related concepts during the process of text comprehension. We also simulate the imposition of external knowledge not explicitly mentioned in the record by using knowledge from the UMLS and the biomedical literature to curate the graph. Finally, we use spreading activation on the graph to simulate the integration component of Kintsch’s model, which resolves inconsistencies by spreading activation across the links between concepts, such that concepts that are contextually consistent will ultimately be more activated.

## Methods

### Sources of data

We used four different data sources: 1. UMLS concepts extracted from the clinical notes in our CDW using MetaMap [13]; 2. A Reflective Random Indexing (RRI) representation of the UMLS concepts [19,20]; 3. The relationships in the 2010 AA UMLS Metathesaurus; 4. A database of semantic predications extracted from the biomedical literature using SemRep [21]. The relationships derived from the metathesaurus and SemRep were used to select conceptual relations that were more likely to be clinically relevant, as RRI alone draws a measure of general relatedness between concepts only.

### Experimental design

Our experiment consisted of four phases: 1. Compute a measure of pairwise correlation between UMLS concepts extracted from the entire CDW; 2. Build a nearest neighbor graph of UMLS concepts based on this pairwise correlation measure; 3. Use the graph to perform inference on a patient-by-patient basis.

### Computing a measure of pairwise correlation between UMLS concepts

We used MetaMap (http://metamap.nlm.nih.gov) [13] to extract UMLS concepts from 1,540,173 clinical notes belonging to outpatient records for 260,772 patients in the UTHealth CDW, after excluding patients in the test dataset (described below). We generated a file for each individual patient containing the UMLS Concept Unique Identifier (CUI) for each UMLS concept identified by MetaMap within that patient’s record, thereby creating a representation of each patient as a "bag of concepts". However, given the large dimensionality of the UMLS (over 2,500,000 unique concepts), a naïve representation of each unique concept as a dimension would be very sparse.

We therefore used Reflective Random Indexing (RRI) [19], a variant of Random Indexing [20] to derive a measure of the similarity between pairs of concepts, as implemented in the Semantic Vectors software package (http://code.google.com/p/semanticvectors/) [22,23]. The random indexing paradigm involves the generation of a *semantic vector* representation of a given concept by superposition of randomly constructed *elemental vector*s representing the contexts in which the concept occurs [22,23]. First, we generated random elemental vectors, 1000-dimensional sparse vectors that are mutually orthogonal or close-to-orthogonal, for each CUI. Then, we generated a semantic vector for each document in the corpus of clinical notes by adding the elemental vector for every CUI extracted by MetaMap from a given document using log-entropy weighting to emphasize CUIs that occur focally in the corpus, and normalized the result. Finally, we built a second set of vectors for each UMLS concept (i.e. CUI) by adding the document vectors for every document in which a given CUI occurs, and normalizing this result by dividing every dimension by the length of the vector.This second set was called the semantic concept vectors.

**Figure 1:**
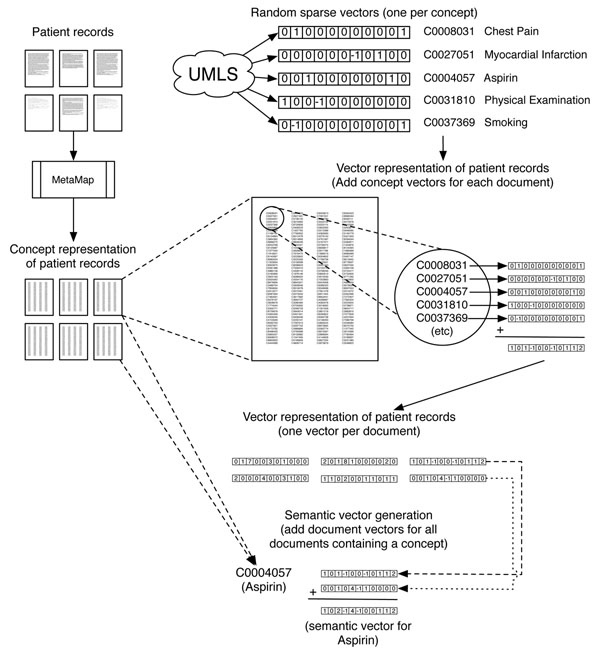
**Simplified RRI workflow** Patient records are turned into a concept representation by MetaMap, which is then used to generate patient vectors. Patient vectors are used to generate semantic vectors for UMLS concepts. Note that the random and semantic vectors can contain real numbers, and are normalized in actual use.

The RRI approach provides a computationally convenient way to capture second-order associations: meaningful estimates of the relatedness between a pair of CUIs can be calculated even if these do not co-occur directly in any patient record. A detailed account of RRI is beyond the scope of this paper, but we refer the interested reader to [19]. For a simplified graphical representation, please see Figure 1.

The semantic vector representation allowed us to compute the relatedness between any pair of CUIs in our CDW by calculating the cosine of the angle between their semantic vector representations. In other words, we computed the Vector Cosine Comparison (VCC) between pairs of vectors. The pairs with the highest VCC were most closely correlated. The advantage of using a semantic vector representation is that their small dimensionality allows a very large number of vectors in RAM simultaneously. The ability to keep the entire vector store in RAM makes the computations extremely fast.

### Constructing a nearest-neighbors graph

We built a nearest-neighbors graph by starting with a seed concept (in this case, C0006142, Malignant neoplasm of breast), then performing a breadth-first search adding the nearest neighborss to the seed (i.e., the concepts with the semantic vectors that had the highest VCC to the seed concept). We recursively iterated through the most closely-related concepts to the seed concept, and added any previously unseen concepts to the graph. We added up to six concepts at each level, and recursed at most three times to limit the size of the graph. In other words, the first level added six concepts; the second level, up to 36 concepts (six for each concept in the first level); the third level, up to 216 (6^3^). If a concept was already present in the graph, it was not added again. We selected the thresholds arbitrarily, based on the observation that human beings have a working memory of between five and nine items [24]. We used the result of the VCC between concepts in the CDW as the weight of the edge.

Nearest-neighbor graphs tend to be noisy as RRI does not capture the nature of the relationship between concepts; we therefore used computable knowledge from the literature and the UMLS to filter the graph. We checked each nearest-neighbor relation against two databases. We removed relations that were absent in both databases from the graph. One database was the UMLS Metathesaurus. Any relationship between two concepts present in the Metathesaurus was enough to validate the nearest-neighbor relationship. The second database was a set of triples (concept-relationship-concept) extracted from the literature by SemRep from a set of 10,000,000 biomedical articles in PubMed. If the relationship was present in either database, it was kept, and labeled with the name of the relationship (either from the UMLS or the literature).

### Perform inference on a patient-by-patient basis

We instantiated and manipulated the graphs with our own MEDRank software (https://github.com/drh-uth/MEDRank, [25]). After instantiating the graph for each patient, every concept was assigned a starting value of 0. We populated each patient’s graph with concepts extracted by MetaMap from that patient’s clinical notes. MetaMap produces a confidence score between 0 and 1000 for each instance of a concept in a given document. We mapped these scores linearly to the range [0,1]. We then added all normalized confidence scores for each concept for each patient. We considered UMLS concepts that were not found in the patient’s record to have a score of 0. For every concept in the graph, we set its starting activation to the sum of its normalized confidence scores in all of a patient’s notes.

After populating the graph for a single patient, we spread activation along the graph's edges for a maximum of three steps. The new activation for each node was the sum of its weighted incoming weights, minus the weight it spread to other nodes. We then read the value of the node corresponding to the original seed concept, and used this as the output of the process.

### Evaluation

We created a test set by taking a random sample of 10,000 records of our CDW. We then eliminated records that did not meet our inclusion criteria (Additional file 1). The remaining 428 records were reviewed by a clinician (PBS), who labeled them as "Breast Cancer" (meaning that the patient had, at some point in his or her life, been diagnosed with any kind breast cancer) or "No breast cancer" meaning that the patient had never been diagnosed with breast cancer.

Our baseline was the performance of MetaMap acting as an automated coding system. We compared this to the performance of our graph-based spreading activation system.

The outcome measure was the ability to discover the state of the patient as determined by the clinician. We counted the number of true positives, true negatives, false positives, and false negatives for the computed state of the patient. We calculated the precision, recall, F1 (harmonic mean of precision and recall), and overall accuracy of the automated process.

By measuring the peformance of MetaMap using this outcome measure with and without the graph-based process, we were able to determine how the graph-based process changed the quality of concept extraction.

## Results

**Figure 2:**
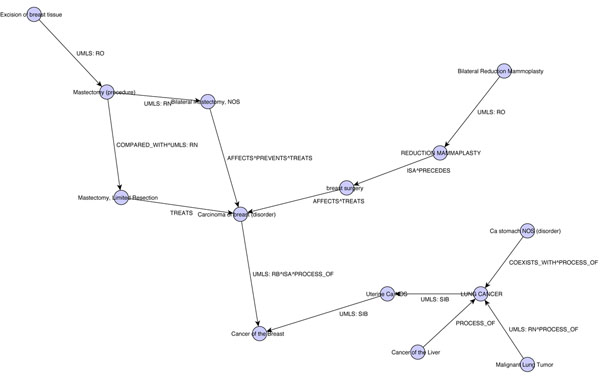
**Graph for Breast Cancer** The graph generated by our iterative process for UMLS Concept C0006142, Breast Cancer. The edge labels specify the type of relationship; relationships prefixed with “UMLS:” were found in the UMLS. Relationships without a prefix (i.e. “PROCESS_OF”) were discovered in the literature.

The resulting graph contains 20 nodes and 18 edges. Of these, six nodes and four edges were not connected to the main body of the graph, and therefore could not influence the output value. Thus, the active graph contained 14 nodes and 14 edges and is presented in Figure 2.

Our test set contained 428 patients. Of these 428 patients, 82 had breast cancer (19.2%) and 346 (80.8%) did not.

MetaMap's overall accuracy at identifying breast cancer patients was 51.1%; its recall was 85.4% (i.e. it found 70 of the 82 patients who had breast cancer), its precision was 26.2%, and its F1 value was 40.1%. The graph-based discovery process labeled 38 patients as positive (recall=46.3%). Its precision was 61.2%, and its F1 was 52.8%. Its overall accuracy was 84.1% (Table 1).

**Table 1:** Performance of MetaMap and the graphical method on determining the breast cancer status of the patient as determined by a physician

	Accuracy	Recall	Precision	F_1_
MetaMap	51%	85%	26%	40%
Graphical method	84%	46%	61%	53%

## Discussion

Our graph-based process complements MetaMap by leveraging structured knowledge (the UMLS) and predicate triples obtained from the literature. It requires no training or manual adjustments, improves overall accuracy and F1. We were able to use graph-based techniques to successfully integrate signals from our clinical records, the UMLS, and the biomedical literature.

To better understand MetaMap’s performance, we examined 10 false positives. In all 10 cases, breast cancer was mentioned in relation to the patient's family history. Unfortunately, developing a reasonable rule-based system to eliminate family-history related false positives is difficult. There are many different ways in which the relationship is described, from "Patient has a sister who may have had breast cancer" to "Family history: Breast cancer -- grandmother." Although we considered removing text labeled “family history” from the clinical notes, the text may be part of a longer narrative section, or may have one of several different, sometimes ambiguous headers. Developing a system to reliably detect family history references is analogous to detecting breast cancer references in the first place. In other words, it is a subtle text classification problem that may require intensive development and fine-tuning at each insititution.

On the other hand, we acknowledge that we are showing merely a proof of concept. While we believe that our technique has great potential, we are presenting only a single condition for which it works.

We have not yet leveraged the different kinds of relationships between concepts in the graph to improve performance. It is possible that TREATS and DIAGNOSES relationships, for example, are much more important than IS_A or parent/child relationships from the UMLS for the purposes of discovering patients with a certain condition.

Our graph construction process was inspired by a high-level understanding of clinician reasoning (i.e. "Does the file say that the patient has breast cancer? If not, does it say that the patient has been treated with procedures commonly used to treat breast cancer?"), but it does not reflect a formal model of diagnosis. The parameters we used in this study were inspired by cognitive models of text comprehension, in which elements of the text and associations from the mind of the reader are integrated, and discrepancies resolved through spreading activation such that the ultimate representation favors those concepts that are contextually appropriate (i.e., mutually consistent).

Additionally, although the completely unsupervised graph-based discovery approach presented here worked well, it may work even better with some human input. For example, Tamoxifen is almost exclusively used to treat breast cancer, and it was not discovered by the graph-building process. We believe that it was not discovered in this study because its strength of association in our CDW is low, due to the fact that our breast oncological practice is small and focused on surgical treatment. It is likely to appear in graphs built at other institutions, and adding it manually to the graph would potentially improve its generalizability.

Although we demonstrate our technique on breast cancer, the methodology will clearly apply to other diseases. In the near future we will undertake the necessary test data set construction to evaluate how well the graph-based technique generalizes to other conditions.

## Conclusions

Graph-based approaches can leverage existing external knowledge to improve concept extraction from clinical text. Unlike previous approaches to this problem, it requires neither the development of customized rules, nor the construction of an expert-annotated training set for supervised machine learning. It outperforms MetaMap when identifying breast cancer patients. Since this approach is disease-independent, it has the potential to generalize to other conditions.

## Authors' contributions

JRH developed the BFS graph generation technique, the idea to use it to classify patients, the software to perform it, and drafted the paper. DS participated in the experimental design, and edited the manuscript extensively. TC created the RRI implementation, the concept-level vector model of the CDW, drafted sections of the paper, and provided extensive guidance on the use of distributional semantic models for this work. PABS and CFB created and labeled the test dataset. CFB also contributed to the writing and editing of the manuscript. EVB participated in the design of the study and techniques, definition of the data set, and revised and edited the manuscript.

## Competing interests

The authors declare that they have no competing interests.

## Supplementary Material

Additional file 1 **Inclusion criteria** Complete list of inclusion criteria for manual review Click here for file

## References

[B1] van der Lei J Use and abuse of computer-stored medical records Methods Inf Med 1991 30 2 79 80 1857252

[B2] Bernstam EV Herskovic JR Reeder P Meric-Bernstam F Oncology research using electronic medical record data American Society of Clinical Oncology, June 4-8 2010. Chicago, IL 2010

[B3] Boyd M Specks U Finkielman JD Accuracy of the ICD-9 code for identification of patients with Wegener's granulomatosis J Rheumatol 2010 37 2 474 10.3899/jrheum.091017 20147488

[B4] Chibnik LB Massarotti EM Costenbader KH Identification and validation of lupus nephritis cases using administrative data Lupus 2010 19 6 741 743 10.1177/0961203309356289 20179167 PMC2964351

[B5] González-Fernández M Gardyn M Wyckoff S Ky PKS Palmer JB Validation of ICD-9 Code 787.2 for identification of individuals with dysphagia from administrative databases Dysphagia 2009 24 4 398 402 10.1007/s00455-009-9216-1 19399554

[B6] Liao KP Cai T Gainer V Goryachev S Zeng-Treitler Q Raychaudhuri S Szolovits P Churchill S Murphy S Kohane I Karlson EW Plenge RM Electronic medical records for discovery research in rheumatoid arthritis Arthritis Care Res 2010 62 8 1120 1127 10.1002/acr.20184 PMC3121049 20235204

[B7] Malik A Dinnella JE Kwoh CK Schumacher HR Poor validation of medical record ICD-9 diagnoses of gout in a veterans affairs database J Rheumatol 2009 36 6 1283 1286 10.3899/jrheum.081195 19447931

[B8] Miller ML Wang MC Accuracy of ICD-9-CM coding of cervical spine fractures: implications for research using administrative databases Ann Adv Automot Med 2008 52 101 105 19026227 PMC3256774

[B9] Singh J Holmgren A Noorbaloochi S Accuracy of Veterans Administration databases for a diagnosis of rheumatoid arthritis Arthritis Care Res 2004 51 6 952 957 10.1002/art.20827 15593102

[B10] Thirumurthi S Chowdhury R Richardson P Abraham NS Validation of ICD-9-CM diagnostic codes for inflammatory bowel disease among veterans Dig. Dis. Sci 2010 55 9 2592 2598 10.1007/s10620-009-1074-z 20033847

[B11] Nahm M Nguyen VD Razzouk E Zhu M Zhang J Distributed cognition artifacts on clinical research data collection forms AMIA Summits Transl Sci Proc 2010 2010 36 40 21347145 PMC3041537

[B12] Stanfill MH Williams M Fenton SH Jenders RA Hersh WR A systematic literature review of automated clinical coding and classification systems J Am Med Inform Assoc 2010 17 6 646 651 10.1136/jamia.2009.001024 20962126 PMC3000748

[B13] Aronson A Effective Mapping of Biomedical Text to the UMLS Metathesaurus: The MetaMap Program AMIA Symposium 2001 17 21 PMC2243666 11825149

[B14] Savova GK Masanz JJ Ogren PV Zheng J Sohn S Kipper-Schuler KC Chute CG Mayo clinical Text Analysis and Knowledge Extraction System (cTAKES): architecture, component evaluation and applications Journal of the American Medical Informatics Association 2010 17 5 507 513 10.1136/jamia.2009.001560 20819853 PMC2995668

[B15] Friedman C Shagina L Lussier Y Hripcsak G Automated encoding of clinical documents based on natural language processing J Am Med Inform Assoc 2004 11 5 392 402 10.1197/jamia.M1552 15187068 PMC516246

[B16] The WEKA data mining software: an update 2009 11 1 10 18

[B17] Automated concept-level information extraction to reduce the need for custom software and rules development 2011 18 5 607 613 10.1136/amiajnl-2011-000183 PMC3168318 21697292

[B18] Kintsch W Comprehension: a paradigm for cognition 1998 Cambridge University Press 461

[B19] Cohen T Schvaneveldt R Widdows D Reflective Random Indexing and indirect inference: a scalable method for discovery of implicit connections J Biomed Inform 2010 43 2 240 256 10.1016/j.jbi.2009.09.003 19761870

[B20] Cohen T Widdows D Empirical distributional semantics: methods and biomedical applications J Biomed Inform 2009 42 2 390 405 10.1016/j.jbi.2009.02.002 19232399 PMC2750802

[B21] Fiszman M Rindflesch T Kilicoglu H Abstraction Summarization for Managing the Biomedical Research Literature Proc HLT NAACL Workshop on Computational Lexical Semantics 2004 76 83 http://dl.acm.org/citation.cfm?id=1596442

[B22] Widdows W Ferraro K Semantic vectors: a scalable open source package and online technology management application Proceedings of the Sixth International Conference on Language Resources and Evaluation (LREC'08) 2008 European Language Resources Association http://www.lrec-conf.org/proceedings/lrec2008/pdf/300_paper.pdf

[B23] Widdows D Cohen T The semantic vectors package: New algorithms and public tools for distributional semantics Proceedings 4th IEEE International Conference on Semantic Computing 2010 Pittsburgh, PA 9 15

[B24] Miller GA The magical number seven, plus or minus two: some limits on our capacity for processing information The Psychological Review 1956 101 343 352 10.1037/0033-295x.101.2.343 8022966

[B25] Herskovic JR Cohen T Subramanian D Iyengar MS Smith JW Bernstam EV MEDRank: Using graph-based concept ranking to index biomedical texts Int J Med Inform 2011 80 6 431 441 10.1016/j.ijmedinf.2011.02.008 21439897 PMC3090689

