# Supplementary material for: Graph-based signal integration for high-throughput phenotyping
Source: BMC Bioinformatics. 2012 Aug 24;13(Suppl 13):S2. doi: 10.1186/1471-2105-13-S13-S2 (PMC3426800; doi:10.1186/1471-2105-13-S13-S2)
Supplement: Additional file 1 — Inclusion criteria Complete list of inclusion criteria for manual review [file 1471-2105-13-S13-S2-S1.docx]

**Appendix**

Inclusion criteria

| Criterion | Accepted values |
| --- | --- |
| String in clinical note | “cancer breast” |
|  | “breast cancer” |
|  | “carcinoma breast” |
|  | “breast carcinoma” |
|  | mastectom* |
|  | lumpectom* |
| ICD-9-CM diagnosis in billing data | 174* |
|  | 175* |
|  | V10.3 |
| Medication | Tamox* |
| Problem in problem list | Adenocarcinoma Of The Breast With Metaplasia |
|  | Adenofibrosarcoma Of The Breast |
|  | Clear Cell Adenocarcinoma Of The Breast |
|  | Malignant Carcinoma Of The Breast |
|  | Mixed Cell Adenocarcinoma Of The Breast |
|  | Mucinous Adenocarcinoma Of The Breast |
|  | Papillary Adenocarcinoma Of The Breast |
|  | Solid Carcinoma Of The Breast |
|  | Tubular Adenocarcinoma Of The Breast |
|  | Alveolar Adenocarcinoma Of The Breast |
|  | Breast Neoplasm Of Uncertain Behavior |
|  | Cystadenocarcinoma Carcinoma Of The Breast |
|  | Infiltrating Duct And Lobular Carcinoma Of The Breast |
|  | Keratinizing Squamous Cell Carcinoma Of The Breast |
|  | Large Cell Neuroendocrine Carcinoma Of The Breast |
|  | Malignant Breast Neoplasm |
|  | Malignant Breast Neoplasm M0 |
|  | Malignant Breast Neoplasm Stage IIB |
|  | Trabecular Adenocarcinoma Of The Breast |
|  | Undifferentiated Carcinoma Of The Breast |
|  | Adenocarcinoma Of The Breast |
|  | Basal Cell Adenocarcinoma Of The Breast |
|  | Infiltrating Duct Carcinoma Of The Breast |
|  | Infiltrating Ductal Carcinoma Of The Breast |
|  | Inflammatory Carcinoma Of The Breast |
|  | Intraductal Papillary Adenocarcinoma Of The Breast With Invasion |
|  | Malignant Breast Neoplasm Stage IV |
|  | Malignant Breast Neoplasm TX |
|  | Medullary Carcinoma Of The Breast |
|  | Acinar Cell Carcinoma Of The Breast |
|  | Breast Cancer |
|  | Comedocarcinoma Carcinoma Of The Breast |
|  | Epithelial-myoepithelial Carcinoma Of The Breast |
|  | Infiltrating Ductular Carcinoma Of The Breast Mixed With Other Types Of Carcinoma |
|  | Lobular Carcinoma Of The Breast |
|  | Malignant Breast Neoplasm Staging |
|  | Malignant Female Breast Neoplasm |
|  | reast Cancer |
|  | Comedocarcinoma Carcinoma Of The Breast |
|  | Epithelial-myoepithelial Carcinoma Of The Breast |

Inclusion criteria (all patients not explicitly included were excluded). A match on any inclusion criteria was enough to flag the patient for manual review. The asterisk (“*”) is a wildcard for searches.
